# Grounded L-Shaped Corner-Embraced Patch Array Polarization Conversion Metasurface Compatible with Multiband and Broadband Characteristics

**DOI:** 10.3390/mi16111240

**Published:** 2025-10-30

**Authors:** Shi Kong, Yifei Zhang, Ziheng Fu, Rui Yang

**Affiliations:** 1 China Airborne Missile Academy, Luoyang 471009, China; kssk2001@163.com; 2 National Key Laboratory of Air-Based Information Perception and Fusion, Luoyang 471009, China; 3 National Key Laboratory of Radar Detection and Sensing, School of Electronic Engineering, Xidian University, Xi’an 710071, China; 24021211458@stu.xidian.edu.cn

**Keywords:** metasurface, polarization conversion, multi-band, broadband

## Abstract

We propose a linear polarization rotator metasurface composed of a grounded array of L-shaped corner-embraced patches. Both simulation and experimental results confirm that the metasurface achieves a polarization conversion ratio exceeding 90% across four distinct frequency bands: 5.7–6.2 GHz, 7.0–8.7 GHz, 11.2–13.9 GHz, and 16.7–18.0 GHz, with near-unity conversion efficiency at four resonance points. Furthermore, the metasurface demonstrates effective polarization conversion over an ultra-wide frequency range from 5.5 to 18.1 GHz. The combination of multiband and broadband characteristics makes this design highly valuable for advanced applications in complex electromagnetic environments, including multifunctional radar systems requiring multiband capabilities and wireless communication systems demanding ultra-wideband performance.

## 1. Introduction

A polarization converter functions to modify the polarization characteristics of electromagnetic waves, allowing the wave to switch from one polarization form to another. According to how it operates, such a device can realize transformations including linear-to-linear [1], linear-to-circular [2], circular-to-linear [3], and even perform both linear-to-linear and linear-to-circular conversions [4], which can take place in either transmission or reflection modes. Recent progress has shown that multilayer metasurfaces, such as tri-layer architectures, are capable of achieving arbitrary spatial polarization distributions, demonstrating their tremendous potential in electromagnetic wave manipulation [5]. Building upon these advances, metasurface-based polarization converters have emerged as a key technology for advanced electromagnetic control owing to their compact configuration, high conversion efficiency, and excellent design flexibility [6,7].

By tailoring the surface impedance at subwavelength scales, these structures enable precise polarization control across a wide range of frequencies. Such manipulation is fundamentally governed by the boundary-condition-based electromagnetic theory of metasurfaces [8]. Among them, linear to-linear polarization conversion metasurfaces are particularly crucial in applications such as radar systems [9,10], wireless communications [11,12], and electromagnetic stealth [13,14], where precise control over polarization orientation is essential for improving signal quality, system adaptability, and anti-interference performance.

To address the demand for multi-tasking and spectrum efficient operation, multiband polarization converters have been developed to support polarization manipulation across multiple discrete frequency bands [15,16]. For instance, the angular stable triple-band in [17] operates within 5.35–5.69, 7.6–8.76, and 12.4–13.96 GHz, while the multi-resonant structure in [18] achieves different polarization modes at 7.3, 9.2, and 12.8 GHz using compound resonator elements. Similarly, the design in [19] combines notched square and circular elements to realize dual-band polarization conversion within 5–9 GHz and 14–16 GHz. However, these existing works predominantly concentrate on conversion functionality within narrow or closely spaced frequency bands, often leading to uneven distribution across the spectrum. Such designs are insufficient for complex, crossband applications like modern radar systems, which simultaneously require medium-range surveillance in the C-band (4–8 GHz), high-resolution imaging in the X-band (8–12 GHz), and fine-grained target recognition in the Ku-band (12–18 GHz). Therefore, achieving widely spaced and uniformly distributed multiband polarization conversion within a single metasurface remains a critical challenge.

In parallel, numerous efforts have focused on achieving broadband polarization conversion to accommodate systems that operate over continuous frequency spans [20,21]. For example, the metasurface in [22], utilizing four metallic resonator layers with multiple substrate layers, achieves broadband linear polarization conversion with an aperture efficiency of 51%. Similarly, the design in [23] employs dual-layer FR-4 substrates and open circular rings to expand the relative bandwidth to 111%. Ref. [24] employed an alternating metal–dielectric stack, optimizing circuit parameters via an equivalent circuit model coupled with a genetic algorithm. The designed metasurfaces demonstrate superior performance: its anti-reflection coating achieves <−20 dB reflection and >−0.17 dB transmission from 203 to 357 GHz. The half-wave plate delivers a 15 dB extinction ratio with 41.6% relative bandwidth from 219 to 334 GHz, and the circular polarizer exhibits 15 dB extinction ratio from 251 to 298 GHz with mechanical tuning capability. Ref. [25] addressed the bandwidth limitation of terahertz reflect arrays by proposing a single-layer stub-loaded resonator with a smooth, nearly linear phase response covering 360°. The focusing reflect array constructed therefrom achieves 3 dB relative bandwidths of 23.3% for TE polarization and 23.9% for TM polarization at 1.00 THz center frequency, with efficiencies exceeding 71%.

The current literature demonstrates significant progress in either multiband or broadband polarization converters, but rarely both within a single device. The fusion of multi-frequency selectivity and broadband coverage within one metasurface platform remains underexplored. To address these research gaps, we propose a linear polarization rotator metasurface composed of a grounded array of L-shaped corner-embraced patches. Both simulation and experimental results confirm that the metasurface achieves a polarization conversion ratio exceeding 90% across four distinct frequency bands: 5.7–6.2 GHz, 7.0–8.7 GHz, 11.2–13.9 GHz, and 16.7–18.0 5 GHz, with near-unity conversion efficiency at four resonance points. Furthermore, the metasurface demonstrates effective polarization conversion over an ultra-wide frequency range from 5.5 to 18.1 GHz.

## 2. Modeling and Results

**Figure 1:**
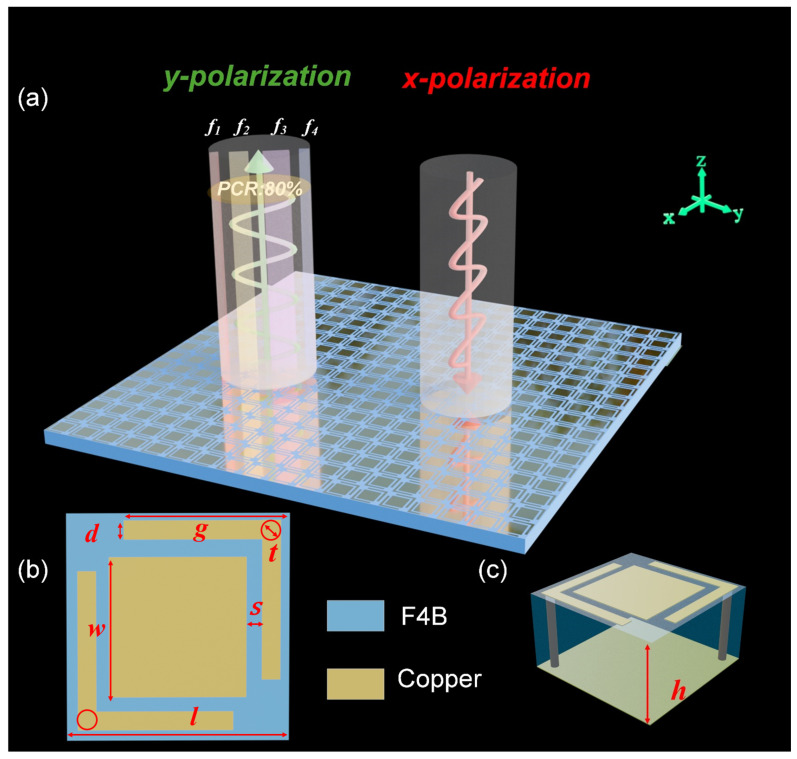
(**a**) Schematic demonstration of the proposed metasurface polarization converter, top view (**b**) and 3D View (**c**) of the unit cell.

As shown in Figure 1, given the x-polarized incidence, the proposed metasurface exhibits four distinct perfect polarization conversion frequencies at $f_{1}$–$f_{4}$, while achieving polarization conversion ratio (PCR) exceeding 80% across the ultra-wideband range. The structure is composed of three layers arranged from top to bottom: a resonant layer, a dielectric spacer, and a reflective ground plane. Both the resonant and reflective layers are fabricated from copper, facilitating strong resonant interactions critical for efficient polarization conversion. The intermediate dielectric layer is constructed from F4B material ($\varepsilon_{r}=2.2$ and tanδ=0.002), with a thickness of h=3.2 mm, optimized to maintain impedance matching and stable polarization conversion across the designated frequency band. The resonant patches incorporate grounded L-shaped hollow corners with through-hole copper pillars, where the inner diameter is defined as *t* = 0.5 mm. The detailed parameter design is listed in Table 1.

To achieve ultra-wideband performance, multiple resonant modes are introduced. The L-shaped corner-embraced patches are specifically designed to excite these modes, supporting multiband polarization conversion. In addition, the inclusion of rectangular patches within the L-shaped corners enhances polarization conversion efficiency while reducing the unit cell size, resulting in a more compact design. The dielectric thickness plays a critical role in controlling phase variation and bandwidth. If the substrate is too thin, strong magnetic coupling between the resonant layer and the ground plane can generate a non-uniform phase gradient, limiting the bandwidth of earlier designs [26]. To mitigate this effect, the dielectric spacer is set to 3.2 mm, and a low-permittivity material is employed to achieve smoother dispersion. Furthermore, unlike other ultra-wideband metasurfaces that rely on multi-layer dielectric stacking, the present design adopts a three-layer structure with ground vias formed by through-hole copper pillars, enabling a reduced thickness and lower-profile configuration.

**Figure 2:**
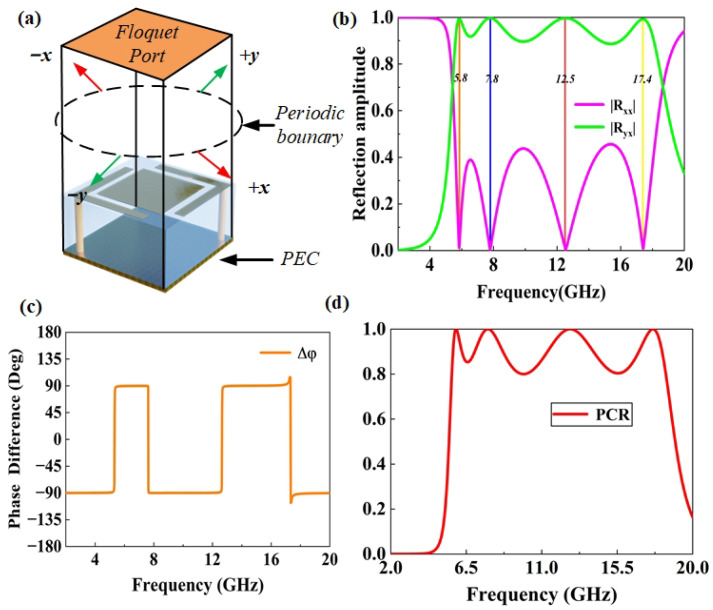
(**a**) Floquet mode analysis. (**b**) Reflection coefficients of co-polarization and cross-polarization, (**c**) Phase difference. (**d**) PCR.

To characterize the polarization conversion behavior of the metasurface, Floquet mode analysis is performed using CST, as shown in Figure 2a. In the simulation setup, periodic boundary conditions are applied along the *x* and *y* directions to emulate an infinitely periodic metasurface array. Along the propagation direction, the upper boundary (+*z*) is defined as an open space, allowing the incident and reflected electromagnetic waves to radiate freely into free space, while the lower boundary (−*z*) is modeled as a perfect electric conductor (PEC) acting as the metallic ground plane. The structure is excited by a normally incident *y*-polarized plane wave, representing the fundamental Floquet mode in the frequency-domain solver. To ensure accurate field resolution, an adaptive meshing strategy is employed, with local mesh refinement automatically applied around metallic edges, gaps, and vias where strong field gradients occur. The average mesh density corresponded to approximately λ/25 at the highest simulated frequency, providing a balance between numerical accuracy and computational efficiency. The minimum element size is limited to 0.5 mm to properly capture subwavelength structural details. Convergence was monitored through the change in S-parameters between consecutive adaptive passes, and the simulation was terminated when the variation fell below −35 dB, guaranteeing stable and reliable numerical results. By applying periodic boundary conditions to the unit cell and using Floquet ports, the structure is excited by a plane wave, and the reflected wave is decomposed into Floquet harmonics. The analysis focuses on the zeroth order reflected modes, where the co-polarized and cross-polarized reflection coefficients are extracted to evaluate polarization conversion efficiency. More specifically, when the *x*-polarized ($E_{i}=\vec{x}e_{xi}$) wave is incident from the top of the metasurface, the reflection transforms into a *y*-polarized wave. The components of the electric field of the incident wave and the reflected wave can be related to each other by the Jones matrix:

(1) $$\left(\begin{array}{c} E_{xr}\\ E_{yr} \end{array}\right)=\left(\begin{array}{cc} R_{xx} & R_{xy}\\ R_{yx} & R_{yy} \end{array}\right)\left(\begin{array}{c} E_{xi}\\ E_{yi} \end{array}\right)$$

Figure 2b shows that the cross-polarization reflection coefficient reaches its maximum at 5.8 GHz, 7.8 GHz, 12.5 GHz, and 17.4 GHz, while the co-polarization component is minimized at these frequencies, indicating resonant behavior and demonstrating near-perfect polarization conversion. As shown in Figure 2c, the phase difference between the co-polarized reflection coefficient $R_{xx}$ and the cross-polarized reflection coefficient $R_{yx}$ remains approximately ±90° across the operating frequency range. This stable phase relationship originates from a dispersion-free polarization conversion mechanism enabled by the metasurface design. Specifically, the grounded L-shaped corner-embraced patches excite hybrid electric and magnetic dipole resonances. The opposite phase slopes of these two resonant responses effectively compensate for each other, resulting in a nearly constant quadrature phase difference between the orthogonal reflected components. Consequently, the metasurface maintains high-efficiency polarization conversion over a broad frequency range, as the electric–magnetic hybrid resonance coupling minimizes phase dispersion and stabilizes the polarization state of the reflected wave. By analyzing the amplitude of the reflection coefficients $\left|R_{xx}\right|$, $\left|R_{yx}\right|$, and phase difference △φ, combined with the Jones matrix, we can further understand how the *x*-polarized wave is converted into a *y*-polarized wave. To evaluate the efficiency of the conversion, we define the PCR. Assuming that the incident wave propagates along the *z*-axis, the PCR is defined as

(2) $$PCR=\frac{{\left|R_{yx}\right|}^{2}}{{\left|R_{yx}\right|}^{2}+{\left|R_{xx}\right|}^{2}}$$

Figure 2d shows that the polarization conversion efficiency exceeds 90% (i.e., the co-polarized reflection parameter S is less than −10 dB) in the frequency ranges of 5.7–6.2 GHz, 7.0–8.7 GHz, 11.2–13.9 GHz, and 16.7–18.0 GHz, and ensures a polarization conversion efficiency greater than 80% over the entire 5.5–18.1 GHz range.

**Figure 3:**
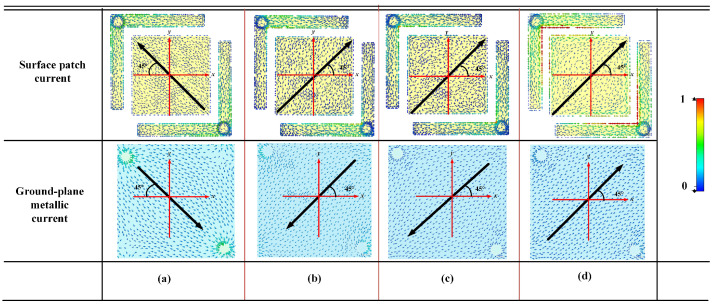
Current distribution at different frequencies (**a**) 5.8 GHz, (**b**) 7.8 GHz, (**c**) 12.5 GHz, (**d**) 17.4 GHz.

To explore the physical mechanism of the proposed polarization converter, we analyze the surface current distribution on the top electromagnetic structure of the unit at four frequencies. When the incident wave is *x*-polarized, an induced current in the *x*-direction is generated. However, due to the structural asymmetry, a portion of this current flows toward the *y*-direction, which induces an electric field along the *y*-direction. When the reflected electric field is orthogonal to the incident electric field and their magnitudes are equal, the angle between the direction of the total surface current induced on the patch and the *x*-axis is 45°, as indicated by the arrow in Figure 3.

As shown in Figure 3, at 5.8 GHz, 7.8 GHz, and 12.5 GHz, the currents on the metallic structure and the ground plane flow in the opposite directions. The image on the top represents the current distribution on the metasurface, while the image on the bottom shows the current distribution on the metallic ground plane. According to Faraday’s law of induction, the opposing current directions between the layers create a time-varying magnetic field, which results in the excitation of magnetic dipoles. This magnetic resonance mechanism contributes to the polarization conversion by enabling the metasurface to interact with the incident wave in a way that modifies the polarization state. At 17.4 GHz, the currents on the metallic structure and the ground plane flow in the same direction. This alignment of current directions indicates the formation of an electric dipole resonance. The coherent alignment of surface currents between the two layers generates an electric field that couples efficiently with the incident wave, thereby facilitating the conversion of the polarization state. The equivalent circuit can also interpret the physical mechanism behind the wideband response and quantitatively describe the resonant behavior. Specifically, the gaps between adjacent metallic patches function as distributed capacitive elements, whereas the patches themselves introduce inductive contributions due to current circulation along their surfaces. Moreover, the elongated conductive paths that link the patches to the grounded vias further enhance the inductive effects, leading to a stronger frequency-selective response. By combining these capacitive and inductive contributions, the overall equivalent circuit can be represented as a series of four coupled LC resonators. Each resonator corresponds to one of the four distinct resonant points revealed in the full-wave simulations, thereby establishing a direct correspondence between the circuit parameters and the observed electromagnetic behavior.

**Figure 4:**
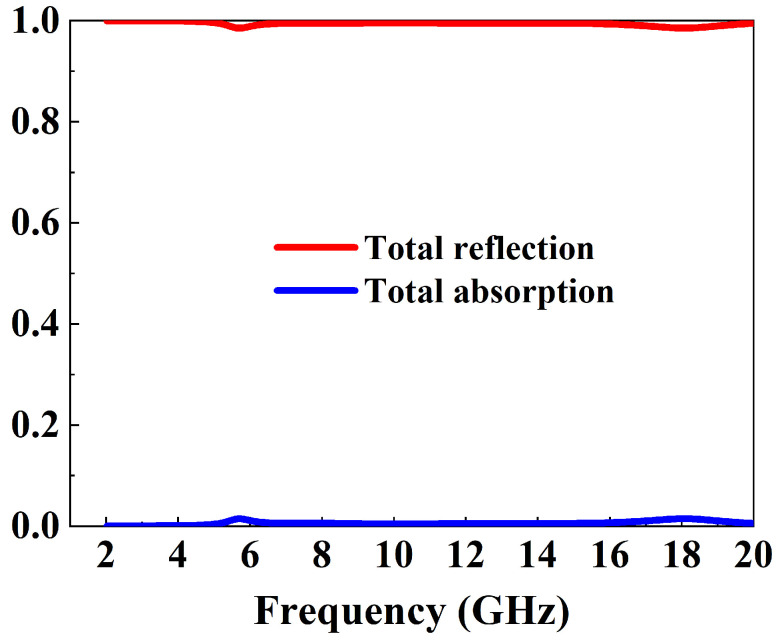
Comparison chart of absorbed and reflected energy.

As shown in Figure 4, it is noted that the sum of $|R_{xx}{|}^{2}$ and $|R_{yx}{|}^{2}$ remains approximately equal to 1 across the entire operating frequency range, indicating that the absorption of the incident electromagnetic wave by the metasurface is extremely low. This observation confirms that almost all of the incident energy is reflected, either in the co-polarized or cross-polarized form, and only a negligible portion is dissipated as dielectric loss. Therefore, when calculating the PCR, the absorption term can be safely neglected without introducing significant error. The minimal absorption demonstrates that the metasurface operates under a nearly lossless condition, ensuring that the calculated PCR accurately represents the true polarization conversion efficiency of the device.

**Figure 5:**
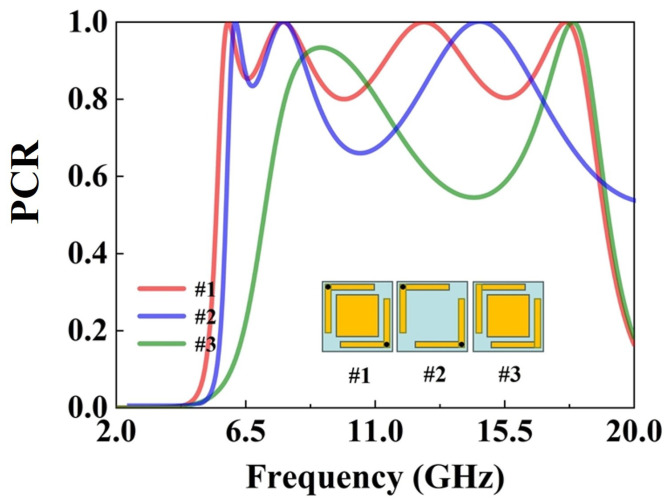
PCR diagram with different unit structures.

To systematically analyze the influence of each structural element, three representative unit cells are selected, as shown in Figure 5. The isolated rectangular patch is not simulated separately because it does not exhibit polarization conversion behavior on its own. Structure 1 includes only the grounded L-shaped corners, Structure 2 combines L-shaped corners with rectangular patches, and Structure 3 further incorporates through-hole copper vias. Comparing these three representative cases helps reveal the individual effects of the rectangular patch and the vias on the polarization conversion performance and bandwidth. As shown in Figure 5, by comparing the proposed metasurface structure with the version that lacks grounding via, we can conclude that incorporating through-hole copper pillars adds the number of resonant points by two and the polarization conversion efficiency is also improved. This is because the introduction of vias connects the upper and lower layers, providing an additional propagation path for electromagnetic energy, so that the electromagnetic energy can not only propagate in the plane but also couple between layers. This multi-path electromagnetic coupling will excite more different resonant modes, thus leading to an increase in the number of resonant points. In addition, by introducing vias to connect the upper and lower layers, a low-impedance transmission path can be constructed. This path can mitigate the impact of parasitic capacitance, enabling more efficient coupling and propagation of electromagnetic energy between layers, thereby improving the polarization conversion efficiency. In addition, when comparing the proposed metasurface structure with the grounded structure containing only L-shaped corners, it can be clearly found that the addition of rectangular patches within the L-shaped corners significantly enhances the polarization conversion effect. This improvement can be attributed to several coupled electromagnetic mechanisms. First, the rectangular patches extend the surface current paths and introduce additional capacitive coupling between adjacent metallic arms, which increases the local electric field intensity around the patch edges. The resulting enhancement in field confinement strengthens the cross-polarized reflection component. Second, the presence of rectangular patches modifies the equivalent LC network of the metasurface unit cell by introducing extra inductive and capacitive branches. These additional resonant pathways enable multiple LC modes to coexist and partially overlap, thereby broadening the effective bandwidth and improving conversion efficiency. Moreover, the compact geometry of the rectangular patches forms shorter current loops and higher current density, enhancing the magnetoelectric coupling and improving the polarization conversion performance without increasing the unit cell size. Collectively, these effects explain the observed enhancement in polarization conversion efficiency and bandwidth after incorporating the rectangular patches.

**Figure 6:**
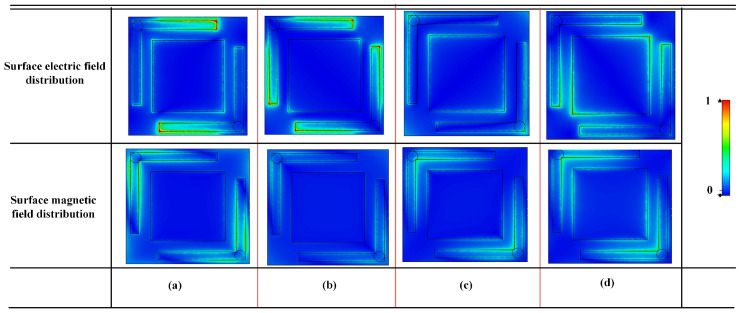
Electromagnetic field distributions at different frequencies (**a**) 5.8 GHz, (**b**) 7.8 GHz, (**c**) 12.5 GHz, (**d**) 17.4 GHz.

By analyzing the surface electric field distribution of the metasurface, it is observed that at 5.8 GHz and 7.8 GHz, the electric field is mainly concentrated on the L-shaped patches, which corresponds well with the results shown in Figure 6. This indicates that at lower frequencies, the diagonally arranged L-shaped patches and their via structures play a dominant role. At 12.5 GHz and 17.8 GHz, the electric field intensity on the rectangular patches gradually increases, which is consistent with the observation in Figure 6. This result demonstrates that the introduction of rectangular patch structures generates additional resonance modes in the higher frequency range, thereby effectively broadening the operating bandwidth. The magnetic field distribution exhibits a similar spatial pattern to that of the electric field, although the overall magnetic field intensity is comparatively weaker.

**Figure 7:**
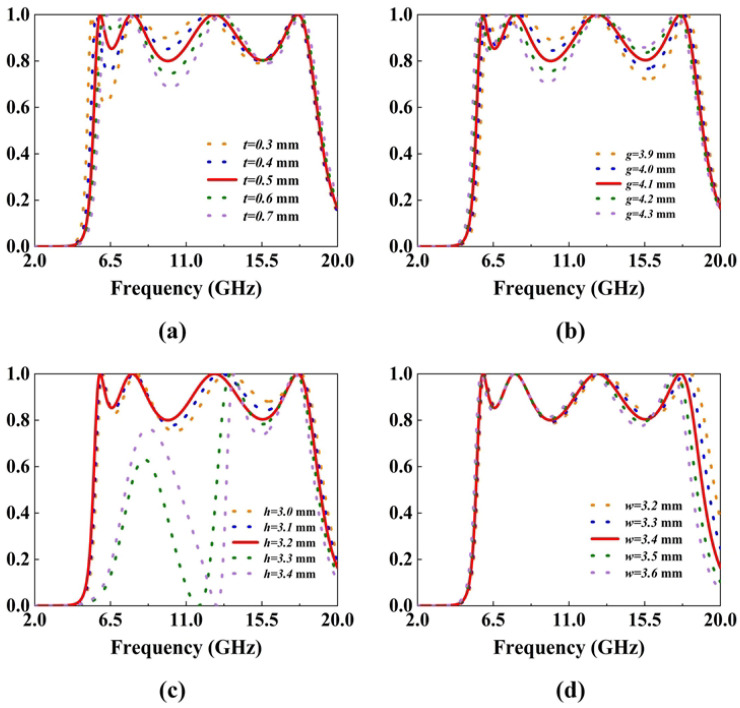
PCR varied with (**a**) t, (**b**) g, (**c**) h, (**d**) w.

Figure 7a illustrates the relationship between the cylinder diameter *t* and the first and second troughs with relatively low conversion rates. As the diameter *t* increases, the conversion rate of the first trough increases significantly. This is because the cylinder determines the characteristics of the low-order modes at low frequencies. The increase in the diameter *t* improves the conversion effect of the first trough. However, if the radius is too large, it will affect the overall structure, resulting in a decrease in the conversion efficiency of the second trough. Figure 7b shows that as the arm length *g* of the L-shape increases, the second frequency point gradually shifts to lower frequencies. This is because a larger *g* extends the current-flowing length of the L-shaped patch, where the equivalent electrical length increases significantly, forcing the redistribution of the surface current. To adapt to the new state of the longer current path, the resonant frequency is adjusted to lower frequencies, showing a redshift. Figure 7c shows that the increase in *h* causes the third frequency point to shift to lower frequencies. This is because *h* extends the physical path of the electromagnetic wave propagation in the vertical direction, and the equivalent electrical length increases significantly. Thus, it changes the surface current distribution and the energy oscillation mode, and finally causes the resonant frequency to shift to lower frequencies. In addition, when *h* is too large exceeding 3.3 mm, the electromagnetic wave will suffer excessive loss during propagation, resulting in the inability to perform polarization conversion at lower frequencies. Figure 7d shows the relationship between the side length *w* of the central rectangular patch and the fourth polarization conversion frequency. As *w* increases, the fourth resonance point gradually shifts toward lower frequencies. This behavior can be attributed to the increased effective electrical length of the central patch, which lowers its resonant frequency. Since the central patch predominantly governs the higher-order mode behavior, its geometric expansion effectively modifies the surface current distribution, resulting in the observed redshift of the fourth polarization conversion band.

**Figure 8:**
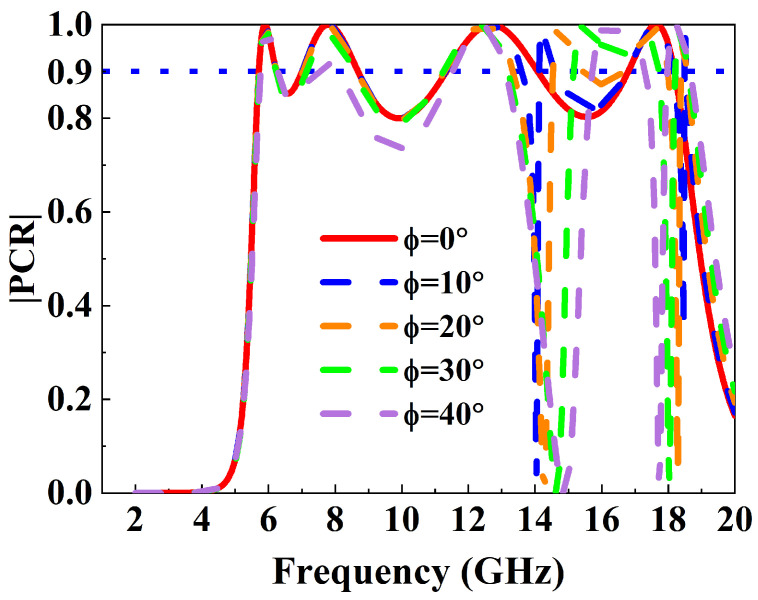
PCR spectrum as a function of incident angle.

To evaluate the practical applicability of the proposed metasurface, we simulated its PCR under oblique incidence angles ranging from 0° to 40° in Figure 8. The results indicate that the metasurface exhibits excellent angular stability in the frequency ranges of 5.5 to 15 GHz and 16 to 17.5 GHz, while it is highly sensitive to the incident angle in the ranges of 13 to 15 GHz and 17.5 to 18.1 GHz. Specifically, the PCR decreases rapidly as the incident angle increases. Clearly, the designed metasurface shows good angular stability in the C-band and X-band. At higher frequencies within the Ku-band, the metasurface performance becomes more sensitive to the incident angle. This degradation mainly results from stronger impedance mismatch between the incident wave and the metasurface, as well as weaker coupling between adjacent unit cells. At larger oblique angles, higher-order resonant and surface modes are excited, which disturb the stable surface current distribution responsible for polarization conversion. In addition, as Φ increases, the projection of the incident electric field on the metasurface plane becomes smaller, reducing the effective excitation of cross-polarized components. These combined effects lead to the reduced polarization conversion efficiency at large incidence angles. Clearly, for stable operation in the higher-frequency Ku-band, more precise control of the incident angle is required.

## 3. Experimental Verification and Comparisons

**Figure 9:**
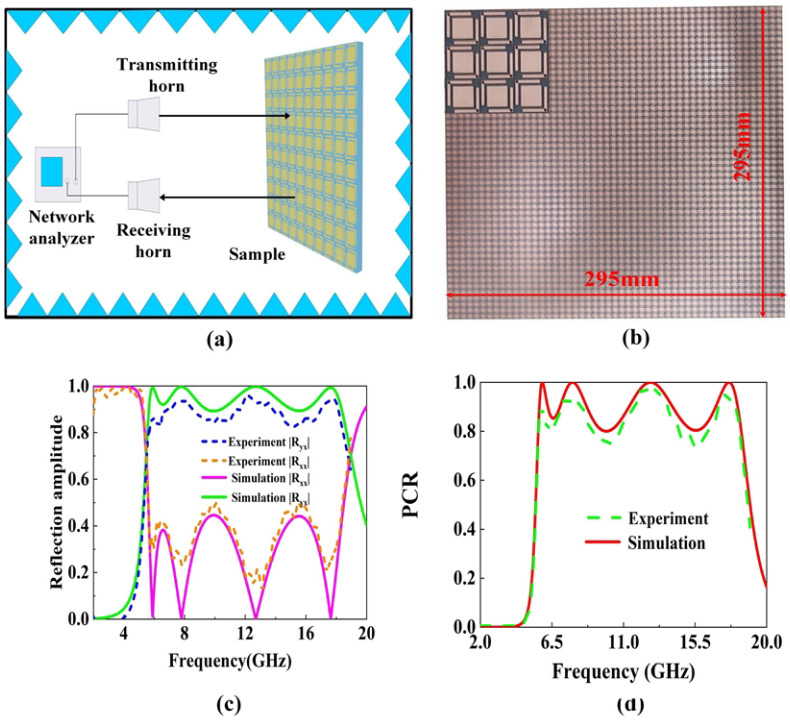
(**a**) Schematic diagram of the experimental testing setup, (**b**) the experiment sample, comparison between simulated and experimental results, (**c**) co-polarized $R_{xx}$ and cross-polarized $R_{yx}$, (**d**) PCR.

As shown in Figure 9a, to experimentally verify the performance of the proposed metasurface, measurements were conducted in a microwave anechoic chamber using an Anritsu MS46322B (Anritsu Corporation, Atsugi, Kanagawa, Japan) vector network analyzer connected to two linearly polarized standard gain horn antennas operating from 1 to 20 GHz. The transmitting and receiving antennas are positioned side by side with a small angular offset to distinguish the reflected signal components. The distance between the antennas and the metasurface sample is adjusted to guarantee the operation in the far-field region. During the measurement, both co-polarized ($R_{xx}$) and cross-polarized ($R_{yx}$) reflection coefficients are recorded across the operating frequency range, and the transmission line losses are calibrated and subtracted from the raw data before processing. All measurements are performed under near-normal incidence in the anechoic environment at room temperature. In the experimental configuration, the proposed polarization converter, as illustrated in Figure 9b, is fabricated into a metasurface composed of a 35 × 35 array of identical unit cells with overall dimensions of 295 mm × 295 mm. Figure 9c,d presents the experimentally measured results alongside full-wave simulation data, including the co-polarized reflection coefficient, cross-polarized reflection coefficient, and the calculated PCR. The experimental results demonstrate a strong correlation with the simulated predictions across the designated operational frequency bands. Within the designated operating frequency band, the experimental results exhibit a strong consistency with the simulated predictions. A slight deviation can be observed between the simulated and measured results, mainly at higher frequencies. This difference arises primarily from fabrication and measurement tolerances. The most sensitive parameters include the cylinder diameter t, the arm length g of the L-shape, dielectric thickness h, and the side length w of the central rectangular patch. As studied in the parameter variation effects on the PCR, we can conclude that non-uniform dielectric thickness alters the coupling between the resonant layer and the ground plane, leading to phase deviations, and etching or milling tolerances in patch width, arm length, and gap size change the equivalent inductance and capacitance of the unit cell, affecting both resonance frequency and amplitude. In addition, small deviations in $\varepsilon_{r}$ (typically ±0.05–0.1) and tanδ can shift the resonance frequencies and reduce the measured PCR. Despite these tolerances, the overall performance confirms the metasurface’s capability for efficient and broadband polarization conversion.

As shown in Table 2, compared with the multiband polarization converters based on arc and cross-shaped patches in [15], the Z-shaped patch design in [16], and the oblique cross pattern in [17], the metasurface proposed in this work offers a greater number of perfect polarization conversion points, which are more uniformly distributed across the frequency bands. Furthermore, in contrast to the multi-layer structural designs reported in [20,23], the introduction of via-grounded structures in the proposed metasurface not only increases the number of resonant points but also enables a low-profile design. In terms of relative bandwidth, bandwidth type richness, and uniform distribution of perfect polarization conversion points, the proposed work outperforms the traditional single-layer structural designs in [27] with multi-arrow patterns and [28] with fork-shaped patterns. In summary, the proposed metasurface realizes efficient polarization conversion over four relatively uniformly distributed frequency bands while maintaining effective polarization conversion over an ultra-wide frequency range. The combination of multiband and broadband characteristics, along with a thinner thickness, makes this design highly valuable for advanced applications in complex electromagnetic environments and more convenient for integration into various compact devices.

## 4. Conclusions

We propose a novel metasurface design that combines multi-frequency conversion and ultra-wideband characteristics. By employing a grounded L-shaped corner-embraced patch array, it achieves perfect polarization conversion at four discrete frequency points (5.8, 7.8, 12.5, and 17.4 GHz), and also demonstrates polarization conversion performance across an ultra-wideband frequency range from 5.5 GHz to 18.1 GHz. Both experimental and simulation results show consistency, validating the feasibility of the design in practical applications. Overall, this work presents an innovative polarization conversion metasurface that integrates multiband and ultra-wideband features. Compared to traditional designs, the proposed metasurface offers more crossband points with more uniform frequency distribution and a thinner overall thickness. In particular, the proposed metasurface can be effectively utilized in multiband radar systems for polarization diversity and clutter suppression. By dynamically converting the polarization of reflected waves across multiple frequency bands, it can improve target detection accuracy and reduce polarization mismatch losses, making it suitable for compact, high-performance radar front-end modules. It has significant practical application potential in fields such as radar system, wireless communication, and electromagnetic stealth.

## Figures and Tables

**Table 1:** Dimensions of the polarization converter.

Parameter	*t*	*w*	*l*	*d*	*s*	*g*	*h*
Value/mm	0.5	3.4	5.8	0.4	0.6	4.2	3.2

**Table 2:** Comparison with other polarization converters.

Reference	Operating Bandwidth (GHz)	Bandwidth Type	Max/Min Relative Bandwidth (%)	Number of Perfect PCR Points	Structural Complexity	Average PCR
[15]	9.4–14.0; 15.5–20.9	Multibands	39.3%/29.7%	3	single-layer substrate structure	>90%
[16]	12.94–16.54; 17.54–26	Multibands	38.9%/24.4%	1	single-layer substrate structure	>90%
[17]	5.35–5.69; 7.60–8.76; 12.41–13.96	Wideband and narrowband coexistence	14.2%/6.2%	3	single-layer substrate structure	>60%
[20]	9.1–18	Ultra-wideband	66%	3	one substrate layers combined with one air layer	>90%
[23]	4–14	Ultra-wideband	111.1%	4	two dielectric substrate layers and one air layer	>90%
[27]	8.0–18.50 GHz	Ultra-wideband	98.4%	3	single-layer substrate structure	>90%
[28]	4.71–5.44; 7.26–9.55; 11.62–12.6; 13.33–13.46	Wideband and ultra-narrowband coexistence	27.2%/0.97%	4	single-layer substrate structure	>80%
This Method	5.5–18.1	Ultra-wideband and multiband coexistence	106.8%/7.5%	4	single-layer substrate structure	wideband > 80%; Multiband > 90%

## Data Availability

The original contributions presented in the study are included in the article, further inquiries can be directed to the corresponding author.
